# Decoding the Link between Periodontitis and Neuroinflammation: The Journey of Bacterial Extracellular Vesicles

**DOI:** 10.2174/0113892029258657231010065320

**Published:** 2023-11-22

**Authors:** Heon-Jin Lee, Youngkyun Lee, Su-Hyung Hong, Jin-Woo Park

**Affiliations:** 1 Department of Microbiology and Immunology, School of Dentistry, Kyungpook National University, Daegu, 41940, Korea, South;; 2 Department of Biochemistry, School of Dentistry, Kyungpook National University, Daegu, 41940, Korea, South;; 3 Department of Periodontology, School of Dentistry, Kyungpook National University, Daegu, 41940, Korea, South

**Keywords:** Periodontitis, neuroinflammatory diseases, alzheimer’s disease, extracellular vesicle, extracellular RNA, oral–brain axis

## 1 INTRODUCTION

A relationship between periodontitis and neuroinflammatory diseases, including Alzheimer’s disease (AD), has long been suspected, considering the increasing number of patients with both conditions with increasing human lifespan [1, 2]. The recent high prevalence of both conditions in the elderly population indicates a strong correlation, with a growing body of research suggesting an “oral microbiome–brain axis.” Periodontitis is an extremely common infectious disease in which oral microorganisms cause inflammation of the periodontal tissues and, in some cases, bone loss; hence, it is not unreasonable to speculate that the same inflammatory factors also cause inflammatory diseases in the brain [3]. It is already well known that oral microbes and microbe-derived molecules can enter the bloodstream because of the high vascularity of the mouth, potentially causing systemic diseases [4]. DNA and RNA from *Porphyromonas gingivalis* (Pg), a bacterium known to cause periodontitis, were recently detected in the postmortem brains of Alzheimer's disease (AD) patients, and animal studies have shown that inhibition of the Pg toxin gingipain can alleviate hallmark features of AD such as amyloid-β deposition and tau tangles [5]. However, there was limited information on how bacteria or bacterial derivatives could reach the brain until Han *et al*. reported that the extracellular vesicles (EVs) of another periodontal pathogen, *Aggregatibacter actinomycetemcomitans*, and their cargo, extracellular RNA (exRNA), could cross the blood–brain barrier (BBB) and reach the brain, promoting an increase in the production of inflammatory factors such as TNF-α in the brains of mice [6]. Subsequently, Ha *et al.* reported that when bacterial EVs were administered through the tail vein, EVs and their exRNA migrated across the meninges to microglia cells in the cerebral cortex as assessed using intravital image analysis [7]. The specific pathway by which bacterial EVs penetrate the BBB is yet to be definitively elucidated. One plausible hypothesis suggests these EVs might utilize transcytosis, a complex, active transport process that orchestrates the vesicular translocation of molecules across the cellular membrane, to achieve this traversal [8, 9]. Nevertheless, this proposition warrants rigorous and comprehensive investigation, particularly in the context of understanding the pathogenesis of bacteria-induced neuroinflammatory diseases.

In fact, EVs are nanosized vesicles, typically measuring 10 - 300 nm in size, and contain a diverse range of cargos, including proteins, nucleic acids, and lipids. EVs are actively secreted by all living cells and play a significant role in interkingdom communications between different biological entities, including bacteria and host cells [10, 11]. “Extracellular vesicles” (EVs), a general term encompassing exosomes, ectosomes, apoptotic bodies, and bacterial outer membrane vesicles (OMVs), is now the recommended nomenclature according to the International Society for Extracellular Vesicles (ISEV), due to the complexities in assigning EVs to specific biogenesis pathways [12].

Emerging studies in the field of gut-brain axis interactions have uncovered that extracellular vesicles, originating from the gastrointestinal bacterium *Helicobacter pylori*, exhibit the ability to migrate to the brain, potentially instigating manifestations associated with Alzheimer's disease [13]. This crucial finding supports the notion that bacterial EVs can in fact traverse to the brain, subsequently triggering neuroinflammation. The phenomenon emphasizes the complex interplay between the human-resident microbiome and the host’s immune response and may have implications for the pathogenesis of neuroinflammatory diseases. The oral cavity harbors more than 700 different species of bacteria, all of which secrete EVs that are constantly produced in saliva and the oral cavity and contain a wide variety of proteins and bacteria-specific metabolites secreted by each bacterium [3, 14]. Therefore, it is not unreasonable to speculate that a similar neural pathway, such as the trigeminal nerve, could potentially facilitate the transmission of oral bacterial EV-mediated symptoms within the oral–brain axis.

Regarding the oral–gut axis, there is a growing body of evidence suggesting that periodontitis can significantly affect a variety of gut symptoms. One remarkable study reported that periodontitis may induce dysbiosis in the gut microbiota, emphasizing the potential systemic implications of oral health [15]. Simultaneously, several other studies have suggested a possible association between oral bacteria and various intestinal diseases [16, 17]. It is believed that this association is due to the proliferation of oral bacteria, which may exacerbate conditions within the gut microbiome. Conversely, recent research has suggested that the gut microbiota influence the development and progression of periodontitis [16], indicating a bidirectional relationship between oral and gut health, where changes in the gut microbiota could manifest in oral health complications, including periodontitis.

Furthermore, bacterial EVs contain large amounts of exRNAs or the so-called microbial-derived small RNAs (msRNAs), which can spread throughout the body [14]. These msRNAs and exRNAs can be recognized by Toll-like receptors (TLRs) in the host, triggering an inflammatory response [18, 19]. TLR7 and TLR8 detect msRNAs in the host, and recent interesting studies have reported that lupus, a type of autoimmune disease, is caused by polymorphic variants of TLR7, which detects viral RNAs, increasing its susceptibility [20], and atherosclerosis, caused by vascular inflammation, is also induced by the activation of TLR8 intracellular signaling by msRNAs and low-density lipoprotein (LDL) [21]. These data strongly suggest that the innate immunity induced by human parasitic bacteria and the host genes involved in immunity are critical for maintaining the homeostasis of the human body [22].

**Fig. (1):**
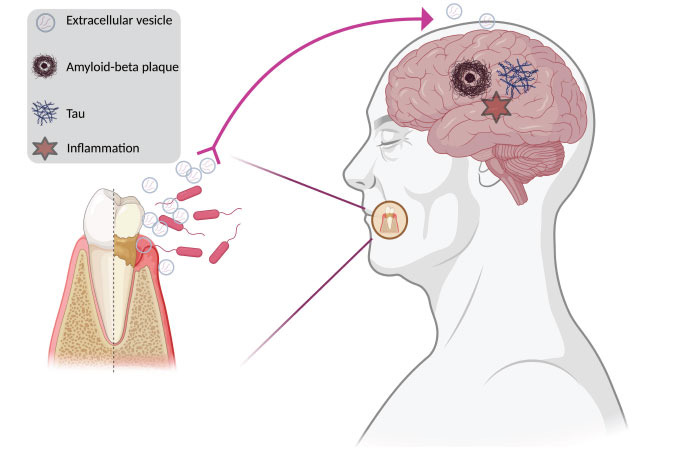
**Oral–brain axis**. Impaired periodontal health could potentially contribute to the onset of neuroinflammatory conditions such as Alzheimer’s disease. This process might be mediated through extracellular vesicles and other derivative products released by oral bacteria, thereby providing a possible link between oral microbial activity and neurological disorders.

Altogether, these findings strongly indicate that EVs and msRNAs from oral bacteria could be transmitted to the brain and promote the development of neuroinflammatory diseases, especially when periodontal health is severely compromised (Fig. **1**). In fact, it is well-known that RNA-seq studies on several patients often yield a significant amount of sequences that are not of human origin [23, 24]. These “junk sequences” may be derived from numerous bacteria in the oral cavity, and the oral bacteria-induced inflammatory response in the brain may be responsible for several diseases. Moreover, the “oral–brain axis” might stimulate further research into how to prevent the entry of pathogen-originated EVs into the host brain. Therefore, it is imperative to decode the msRNAs harbored within bacterial EVs and trace their presence in the human brain utilizing dual RNA-seq (concurrent transcriptomic analysis of both bacterial pathogens and their associated eukaryotic host cells), as they may potentially contribute to the etiology of specific neuroinflammatory diseases. In addition, it is extremely important to elucidate variants in various genes associated with innate immunity that augment susceptibility to bacterial byproducts.

## CONCLUSION

Ultimately, through comprehensive characterization of the bacterial microbiome within bacterial EVs, we assert that salivary exRNAs or msRNAs could be promising, easily accessible biomarkers for early diagnosis of neuroinflammatory diseases due to the non-invasive nature of saliva collection and the oral cavity's proximity to the brain.

## LIST OF ABBREVIATIONS

AD: Alzheimer’s Disease
BBB: Blood–brain Barrier
EVs: Extracellular Vesicles
msRNAs: microbial-derived small RNAs
OMVs: Outer Membrane Vesicles
TLRs: Toll-like Receptors
